# High frequency of Nichols-like strains and increased levels of macrolide resistance in *Treponema pallidum* in clinical samples from Buenos Aires, Argentina

**DOI:** 10.1038/s41598-022-20410-5

**Published:** 2022-09-29

**Authors:** Nicolas Morando, Eliška Vrbová, Asunta Melgar, Roberto Daniel Rabinovich, David Šmajs, María A. Pando

**Affiliations:** 1 CONICET-Universidad de Buenos Aires, Instituto de Investigaciones Biomédicas en Retrovirus y Sida (INBIRS), Buenos Aires, Argentina; 2 Department of Biology, Faculty of Medicine, Masaryk University, Brno, Czech Republic; 3 Programa de Enfermedades de Transmisión Sexual (PETS), Hospital de Clínicas “José de San Martín”, Universidad de Buenos Aires, Buenos Aires, Argentina

**Keywords:** DNA sequencing, Bacterial infection, DNA sequencing, Haplotypes, Bacterial genetics

## Abstract

Globally, 94% of *Treponema pallidum* subsp. *pallidum* (TPA) clinical strains belong to the SS14-like group and 6% to the Nichols-like group, with a prevalence of macrolide resistance of 90%. Our goal was to determine whether local TPA strain distribution and macrolide resistance frequency have changed significantly since our last report, which revealed that Buenos Aires had a high frequency of Nichols-like strains (27%) and low levels of macrolide resistance (14%). Swab samples from patients with suspected syphilis were collected during 2015–2019 and loci TP0136, TP0548, TP0705 were sequenced in order to perform multilocus sequence typing. Strains were classified as Nichols-like or SS14-like. The presence of macrolide resistance-associated mutations was determined by examination of the 23S rDNA gene sequence. Of 46 typeable samples, 37% were classified as Nichols-like and 63% as SS14-like. Macrolide resistance prevalence was 45.7%. Seven allelic profiles were found, five were SS14-like and two were Nichols-like. The frequency of Nichols-like strains increased between studies (26.8% vs. 37%, *p* = 0.36). A dramatic increase was found in the frequency of macrolide resistant strains between studies (14.3% vs. 45.7%, *p* = 0.005). Our results are in agreement with international trends and underscore the need to pursue further TPA molecular typing studies in South America.

## Introduction

*Treponema pallidum* subsp. *pallidum* (TPA) is the bacterial pathogen that causes syphilis, a chronic venereal disease in humans^1^. Despite the availability of several diagnostic tools as well as the effective treatment with penicillin, syphilis prevalence and incidence remain high around the world. The World Health Organization estimated that approximately 22.3 million people globally between the ages of 15 and 49 had syphilis in 2020, with 7.1 million new syphilis cases per year globally, and 1.1 million new cases in the Region of the Americas^2^.

In Argentina, syphilis incidence has been steadily on the rise since 2010, with an increase from 21.2 new cases per 100,000 habitants in 2015 to 56.1 per 100,000 habitants in 2019, and 1.55 cases of congenital syphilis per 1000 live births in 2019^3^. Several epidemiological studies have identified groups that are disproportionally vulnerable to syphilis, such as men who have sex with men, female sex workers and male to female transgender people, with syphilis prevalences between 17 and 50%^4–7^.

The molecular characterization of TPA has been of increasing interest in recent years as a useful tool for improving understanding of syphilis network transmission and epidemiology dynamics. Whole genome sequencing (WGS) of clinical strains from around the world reveals that the global syphilis population comprises just two deeply branching lineages, Nichols and SS14, each with multiple sublineages. The dominant TPA sublineages circulating today are believed to be the result of a rapid population expansion in the 2000s, following a population bottleneck in the 1990s^8,9^. The first typing scheme was designed by the Center for Disease Control and Prevention (CDC) in 1998^10^ and subsequently improved^11,12^. An alternative sequencing-based molecular typing (SBMT) scheme was introduced in 2006^13^ and recently enhanced^14^. The latest multilocus sequence typing (MLST) scheme analyzes the TP0136, TP0548 and TP0705 loci and provides a simple alternative to WGS, retaining around 30% of WGS’s resolution power. Additionally, MLST allows for the distinction between the two TPA clades, SS14 and Nichols (based on TP0136 and TP0548 sequences), as well as the differentiation of strains within each clade (based on TP0136, TP0548 and TP0705 sequences). MLST also has the advantage of distinguishing TPA from other treponemes such as *Treponema pallidum* subsp. *pertenue* and *Treponema pallidum* subsp. *endemicum*^14^. MLST of TPA strains has been expanding since its proposal: the TPA PubMLST database^15^ currently contains 1572 strain records from 40 different countries. However, over 70% of records come from Europe. Both SBMT and MLST can be paired with the analysis of 23S rDNA to determine the presence of mutations associated with macrolide resistance.

We have previously conducted the first characterization of circulating TPA strains in Buenos Aires, Argentina using SBMT, in samples collected between 2006 and 2013. We detected the presence of nine distinct allelic profiles, five of which had not been described before. Out of these nine haplotypes, 77% were related to SS14 and 23% to Nichols strains^16^. These results suggested that the syphilis epidemic in Argentina differed considerably from the global distribution pattern, according to which Nichols-like strains are much rarer^17^.

Even though penicillin is the antibiotic of choice for the treatment of syphilis, there are other antibiotics which can be prescribed in certain situations (e.g. penicillin allergy). Macrolides in particular are a desirable treatment option because of their oral administration, which makes them more readily acceptable by patients. Two point mutations in the 23S rDNA locus (A2058G and A2059G) have been linked to macrolide resistance in TPA strains^18^. Global prevalence of macrolide resistance mutations is 90%, with wide regional variations: Cuba, the US and several European countries report high prevalence rates (61–100%), whereas Canada, Russia, South Africa, Madagascar, Taiwan and Peru report little to no macrolide resistance (0–12%). Our first study seemed to indicate that the situation in Argentina was closer to the latter, with 14% prevalence of macrolide resistance mutations^16^.

Several studies have shown that both the predominant TPA strains and the frequency of mutations associated with macrolide resistance in a particular population can change over time^12,19,20^. With the aim of studying the dynamics of TPA strains in Buenos Aires, Argentina, the purpose of the present study was to characterize the syphilis epidemic in Buenos Aires by analyzing TPA clinical strains collected during 2015–2019 in terms of their strain and haplotype distribution as well as the presence of antibiotic resistance-associated mutations, in order to update and expand on our previous report using the newer MLST methodology.

## Results

### Characteristics of patients and clinical samples

**Figure 1:**
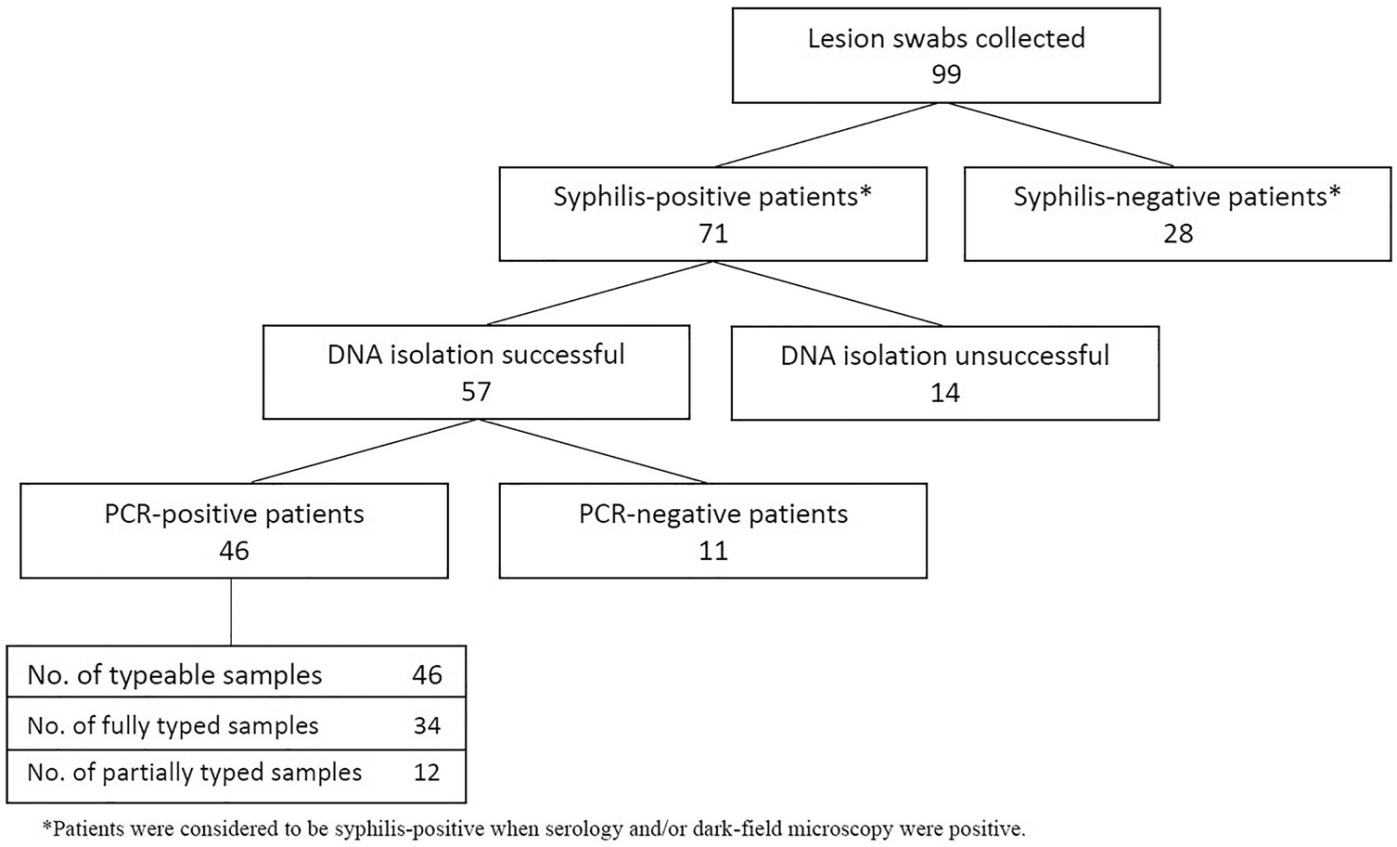
Flow chart of samples collected and analyzed in this study.

A total of 99 swabs were collected from patients with suspected syphilis lesions (Fig. 1). In 28 cases, active syphilis was ruled out due to negative serology (non reactive Venereal Disease Research Laboratory –VDRL– test), which was also confirmed by negative PCR in all cases. Among syphilis-positive patients, DNA isolation was unsuccessful in 14 cases, as evidenced by negative beta-actin PCR. Treponemal DNA (*polA* and/or *tmpC*) amplification was unsuccessful in 11 cases.

Clinical characteristics of patients with syphilis diagnosis (based on serology and dark-field microscopy –DFM– results) and successful DNA isolation are presented in Table 1. Briefly, 83% of patients were men with a median age of 25 years and 46% of those with known HIV status were HIV-positive. Most patients were Argentine nationals (74%) and unmarried (88%), with approximately half of them residing in the city of Buenos Aires (52%) or the Province of Buenos Aires (48%); 52% were students or unemployed. Lesions were mainly located in the genitals (53%), some were oral (28%) and only one was an anal lesion. Most patients (83%) had positive VDRL serology, 65% with high titres (> 1:32). Although data on syphilis stage could not be recovered for almost half of the patients, among those with a determined clinical stage, 45% were primary syphilis cases and 55% were secondary syphilis cases. No significant differences were found between patients with PCR-positive and PCR-negative samples in terms of sociodemographic or clinical characteristics.

**Table 1:** Characteristics of 57 syphilis-positive patients with successful DNA isolation enrolled in this study during 2015–2019 in Buenos Aires, Argentina.

Clinical characteristics of patients	All patients (n = 57)^a^	Patients with PCR-positive samples (n = 46)^a^	Patients with PCR-negative samples (n = 11)^a^
Gender, n (%)	M 47 (82.5); W 10 (17.5)	M 37 (80.4); W 9 (19.6)	M 10 (90.9); W 1 (9.1)
Median age (men/women)	25 (16–69) / 27 (18–35)	25 (16–59) / 26 (18–35)	29 (16–69) / 27
HIV positive^b^, n (%)	10/22 (45.5)	9/20 (45)	1/2 (50)
DFM^c^ (%)	10 P (17.5); 38 N (66.7); 9 n.d. (15.8)	9 P (19.6); 31 N (67.4); 6 n.d. (13.0)	1 P (9.1); 7 N (63.6); 3 n.d. (27.3)
**Serology**			
FTA-ABS/TPHA^d^ (%)	20 P (35.1); 37 N/n.d. (64.9)	17 P (37); 29 N/n.d. (63)	3 P (27.3); 8 N/n.d. (72.7)
VDRL (%)	47 P (82.5); 9 N (15.8); 1 n.d. (1.8)	37 P (80.4); 8 N (17.4); 1 n.d. (2.2)	10 P (90.9); 1 N (9.1); 0 n.d. (0)
≥ 1:32	37 (64.9)	29 (63)	9 (81.8)
**Syphilis stage (%)**			
Primary	13 (22.8)	12 (26.1)	1 (9.1)
Secondary	16 (28.1)	14 (30.4)	2 (18.2)
Undetermined	28 (49.1)	20 (43.5)	8 (72.7)

Lesion type	Samples (n = 57)	Samples (n = 46)	Samples (n = 11)
Genital	30 (52.6%)	25 (54.3%)	5 (45.5%)
Oral	16 (28.1%)	12 (26.1%)	4 (36.4%)
Anal	1 (1.8%)	1 (2.2%)	0 (0)
Oral-genital	1 (1.8%)	1 (2.2%)	0 (0)
Undetermined	9 (15.8%)	7 (15.2%)	2 (18.2%)

^a^*M* men, *W* women, *P* positive, *N* negative, *n.d.* not determined.

^b^HIV status was known only in 22 patients (38.6%).

^c^DFM, dark-field microscopy.

^d^*FTA-ABS* Fluorescent Treponemal Antibody Absorption, *TPHA* *Treponema pallidum* Particle Hemagglutination Assay.

### Multilocus sequence typing (MLST)

All 46 PCR-positive samples were fully or partially typeable using MLST. Samples were considered typeable when at least one sequence of a typing locus (either TP0136, TP0548 or TP0705) was obtained. By comparing the TP0136 and/or TP0548 sequences from our samples with reference sequences we determined that 63% (29/46) of strains belonged to the SS14-like group and 37% (17/46) to the Nichols-like group. In all cases where both TP0136 and TP0548 were successfully sequenced (n = 37), clade assignment was consistent between loci.

**Figure 2:**
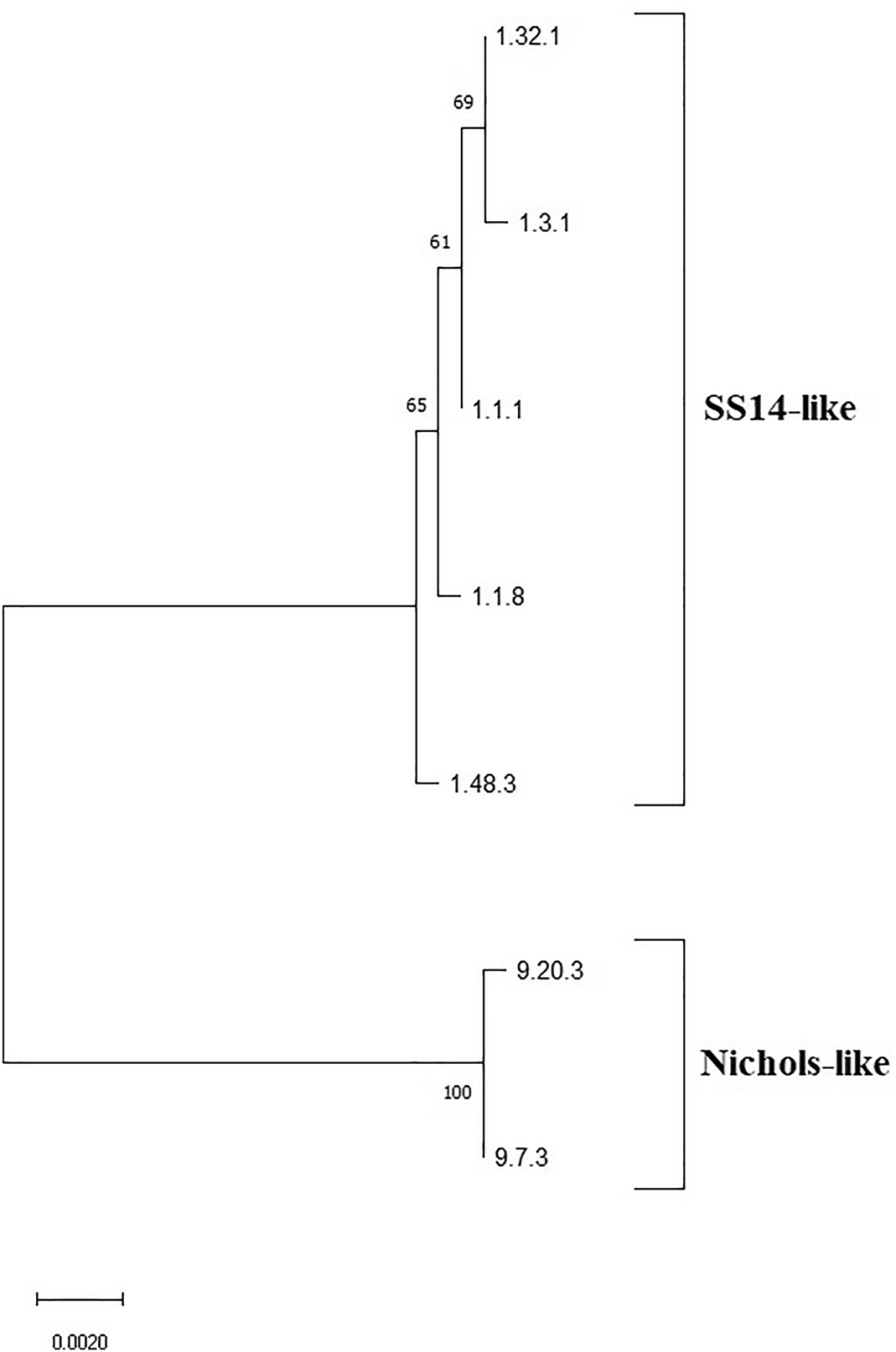
Majority rule consensus tree of 1000 bootstrap replicates performed on concatenated TP0136, TP0548 and TP0705 sequences identified among fully typed samples. Bootstrap percentages given for clades recovered with more than 50% support. The scale shows the number of substitutions per site. Bootstrap values are shown next to branches. The length of concatenated sequences was 1901 bp and contained 44 variable positions. The tree was constructed in MEGA X^52^ using the Maximum Likelihood method^54^ with the bootstrap test^55^.

Samples from 34 (73.9%) patients were completely typed (including the TP0136, TP0548 and TP0705 gene sequences), 29 also including the 23S rDNA gene sequence. Altogether, seven different TPA haplotypes were found among the fully typed: five were related to the sequence from the TPA strain SS14 (1.1.1, 1.3.1, 1.1.8, 1.32.1 and 1.48.3) and two were related to the sequence from the TPA strain Nichols (9.7.3 and 9.20.3). All identified haplotypes and their frequencies are shown in Table 2. Briefly, the most frequent allelic profile was 9.7.3 (35.3% among fully typed samples) followed by 1.1.1 and 1.3.1 (each 26.5% among fully typed samples), and finally by 1.1.8, 1.32.1, 1.48.3 and 9.20.3 with only one case each. Among the partially typed samples, only one SS14-like strain showed an haplotype that was not consistent with any of the fully typed profiles (X.1.3) All alleles found in this study have been described previously; however, we describe the new allelic profile 1.48.3. A phylogenetic analysis of allelic profiles found among fully typed samples is shown in Fig. 2.

**Table 2:** MLST allelic profiles for 34 fully typed and 12 partially typed lesion swab samples from patients attending a sexually transmitted infections clinic in Buenos Aires, 2015–2019.

Sequence type^a^	Allelic profile^b^	Typing	23S rDNA^c^ (no. of samples)	Genetic group	No. of samples (%)^d^
26	9.7.3	Complete	S(7)/R8(2)/X(3)	Nichols	12 (26.1)
2	1.1.1	Complete	S(6)/R8(2)/X(1)	SS14	9 (19.6)
1	1.3.1	Complete	S(1)/R8(8)	SS14	9 (19.6)
31	9.20.3	Complete	R9 (1)	Nichols	1 (2.2)
3	1.1.8	Complete	X(1)	SS14	1 (2.2)
50	1.32.1	Complete	R8(1)	SS14	1 (2.2)
117^e^	1.48.3^e^	Complete	S(1)	SS14	1 (2.2)
–	1.X.1	Partial	R8(1)/X(1)	SS14	2 (4.3)
–	1.X.X	Partial	X(2)	SS14	2 (4.3)
–	9.7.X	Partial	S(2)	Nichols	2 (4.3)
–	X.1.1	Partial	X(1)	SS14	1 (2.2)
–	X.1.3	Partial	S(1)	SS14	1 (2.2)
–	X.3.1	Partial	R8(1)	SS14	1 (2.2)
–	X.3.X	Partial	X(1)	SS14	1 (2.2)
–	9.20.X	Partial	S(1)	Nichols	1 (2.2)
–	9.X.X	Partial	X(1)	Nichols	1 (2.2)

^a^According to the PubMLST database of *Treponema pallidum* subsp. *pallidum*^15^.

^b^Allelic profiles are based on a three-number code: the first number corresponds to the allelic variant in the TP0136 locus, the second corresponds to the allelic variant in the TP0548 locus and the third corresponds to the allelic variant in the TP0705 locus. We used X to denote that the allelic variant was not determined.

^c^*S* no mutation in 23S rDNA (sensitive), *R8* A2058G mutation in 23S rDNA (resistance), *R9* A2059G mutation in 23S rDNA (resistance), *X* not determined. The frequency (number) of each case is given in parenthesis.

^d^Percentages calculated over the total number of typed samples (no. of fully typed samples + no. of partially typed samples).

^e^Newly identified allelic profile, with alleles known from previous studies.

### Prevalence of mutations associated with macrolide resistance

The 23S rDNA gene locus was amplified in 35 samples, and in 16 of them (45.7%), either the A2058G (15 patients, 93.7%) or the A2059G (1 patient, 6.3%) mutation was found. No strains harbored both A2058G and A2059G mutations. The sequences of both copies of the 23S rRNA gene were identical in all cases. Macrolide resistance was detected in both SS14-like (13/22) and Nichols-like (3/13) strains. The frequency of resistance was higher in the SS14 clade, however without statistical significance (59.1% vs. 23.1%, *p* = 0.08).

### Associations of clade and allelic profiles with patient characteristics

We found no statistically significant associations of clade or allelic profile with patient age, gender, residence, nationality, occupation, marital status, sexually transmitted infection (STI) history, location of lesion, VDRL titer, DFM results, syphilis stage or HIV status. Patients carrying Nichols-like strains tended to be slightly older than patients carrying SS14-like strains (median age: 31 vs. 25 years, *p* = 0.07) and to have higher VDRL titers (median titer: 128 vs. 32, *p* = 0.09).

## Discussion

At the local level, this study is the second to analyze the genetic characteristics of circulating TPA in Argentina, serving as both a continuation and an update of the previous report by our research group^16^. While the first study used the SBMT methodology, this study is the first to apply the enhanced MLST scheme in Argentina. At the regional level, this study constitutes as well one of the first initiatives to characterize the diversity of TPA in South America, where only a few molecular epidemiology studies of TPA have been conducted^21,22^, and even fewer using the MLST methodology^23^.

In this study, as in most reports worldwide, the SS14 clade was predominant over the Nichols clade. However, the frequency of Nichols-like strains observed (37%) is considerably higher than is usually the case globally. Clade SS14 is reported to constitute the vast majority (94.1%) of worldwide TPA clinical strains, though the relative frequencies of these clades show some geographical variation: while in most countries the prevalence of Nichols-like strains is lower than 10%, this has been found to be 15% in Japan^24^, 21.4% in Peru, 20.2% in Taiwan and 100% in Madagascar^25^. In Argentina, our first study^16^ showed a frequency of Nichols-like strains of 26.8%, which would make Argentina the country with the highest reported frequency at the time after Madagascar. In the present study we found an even higher prevalence of the Nichols clade which might indicate that this prevalence is currently on the rise. Moreover, our results, together with those of the report from Peru^22^, suggest that South America is a region with a high frequency of Nichols-like circulating strains, though admittedly more data is required to support this claim. However, it must be noted that, although the city of Buenos Aires and its surroundings account for a considerable percentage of the Argentine population, our results may not reflect TPA diversity in other parts of the country.

The explanation for the higher frequency of Nichols-like strains observed in some countries remains elusive, considering that genomic comparisons of these strains do not reveal differences in genes known to confer a fitness advantage^8^. A higher frequency of macrolide resistance in SS14-like strains, like we observed in this study, might explain its overall predominance, but their expansion due to other selective advantages cannot be excluded.

Among fully typed samples, we found seven distinct haplotypes, five of which were related to the SS14 strain (1.1.1, 1.3.1, 1.1.8, 1.32.1 and 1.48.3), while the other two were related to the Nichols strain (9.7.3 and 9.20.3). Haplotypes 9.7.3 (35%), 1.1.1 (27%) and 1.3.1 (27%) accounted for most samples. In our previous study, a different SBMT nomenclature was used and also that study did not include the TP0705 locus, making comparisons difficult. However, a rough comparison clearly reveals that the most common haplotypes have changed. In our previous study, the SS14-like strains SSS, SSR9 and SSR8 (now named 1.1.X) constituted the most prevalent (43.9%), followed by the Nichols-like strains U3U6S and U3U6R8 (now named 9.7.X) representing 17% of the cases. However, in this new study, strain 9.7.X (specifically 9.7.3) constitutes the most prevalent (35.3%) followed by strain 1.1.X (29.4%, including 1.1.1., 26.5%, and 1.1.8, 2.9%) and strain 1.3.X (specifically 1.3.1., 26.5%). Thus, although SS14-like strains as a whole remained dominant, the most prevalent allelic profile changed from an SS14-related haplotype to a Nichols-related haplotype, reflecting the increase in the overall frequency of Nichols strains. The difference in haplotype distribution between the Argentinean studies may be due to the fact that samples were collected at different times (2006–2013 vs. 2015–2019); several studies show that TPA strains in a particular population can vary over time^12,19,20^. Although both studies were aimed at the same population, recruited at the same clinic, differences in TPA diversity may also be partially explained by variations in demographic and clinical characteristics: compared to the previous study, the results presented here correspond to a sample that includes more women (female percentage: 19.6% vs. 4.7%, *p* = 0.03) and less primary syphilis cases (percentage of primary cases among those with determined stage: 46.2% vs. 73.7%, *p* = 0.03).

A comparison of the frequencies of TPA allelic profiles observed in this study with the average frequencies reported in other continents (Table 3) shows that, not only are the relative frequencies of the SS14 and Nichols clades observed in Argentina unusual, but so are the allelic profiles detected. Haplotype 9.7.3, which was found in 35% of our samples, is only present in less than 5% of samples in other continents. Meanwhile haplotype 1.3.1, which accounts for roughly half of clinical samples in Europe and 60% of samples in North America, was found in approximately a fourth of Argentinian samples. Haplotype 1.1.1, though not rare in other continents, was detected in our samples with a considerably higher frequency than usually reported^15,26^. These differences underscore the need for further TPA molecular typing studies in South America, since it cannot be assumed that clade and allelic profile frequencies among local clinical samples will be similar to those in other regions.

**Table 3:** Comparison of reported TPA allelic profile frequencies between this study and studies conducted in other continents.

	1.3.1	1.1.1	9.7.3	1.1.8	9.20.3	1.32.1	1.48.3
Argentina (this study) (%)	26.5	26.5	35.3	2.9	2.9	2.9	2.9
Europe^a^ (%)	48.9	15.1	4.4	7.7	0.15	0.15	0
North America^a^ (%)	60	9.6	2.2	0.74	0	0	0
Asia^a^ (%)	3.9	3.9	0.8	75.8	0	0	0
Total^a^ (%)	44.2	12.8	3.5	15.9	0.11	0.11	0

^a^Data according to the PubMLST database^15^. Only the frequencies of fully typed strains were used for calculations.

Considering that the nucleotide diversity has been demonstrated to be higher within the strains belonging to the Nichols clade than within the strains belonging to the SS14 clade (π = 0.05 for Nichols and π = 0.01 for SS14^17^), and given the higher frequency of Nichols-like strains in our samples, one might expect a higher number of different Nichols-like haplotypes than were found in this study. It must be noted however, that only a limited number of positive samples (46) and detected haplotypes (seven) were obtained, thus it is possible that a lower number of Nichols haplotypes was detected by chance. At the same time, it has been reported that the frequency of some genotypes expands or decreases over time^19,27^.

The observed prevalence of macrolide resistance-associated mutations in the present study was nearly 50%, the A2058G mutation accounting for all but one of these cases. The presence of this mutation was detected in SS14-like as well as Nichols-like strains, with a tendency towards higher mutation frequency among SS14-like strains (59% vs. 23%), which was also the case in our first study^16^. The frequency of macrolide resistance has tripled between our first study and this study (14.3% vs 45.7%, *p* < 0.005) and it places Argentina closer to other countries with high prevalence of macrolide resistance such as Australia^28^, Cuba^27^, Czech Republic^19,23^, China^29–32^, Ireland^33,34^, Spain^35^, the UK^36^ and the US^11,37–39^. As shown in Table 4, even though the presence of macrolide resistance mutations increased with time in both clades, this increase showed statistical significance only in the SS14 clade. The observed increase in the frequency of macrolide-resistant TPA strains in our study population coincides with the global trend^40^.

**Table 4:** Comparison of clade and macrolide resistance frequencies between studies.

	Gallo Vaulet ML, 2017	This study	*p*-value
**Clade**			
SS14	73.2% (30/41)	63% (29/46)	
Nichols	26.8% (11/41)	37% (17/46)	0.36
**Macrolide resistance mutations**			
All	14.3% (6/42)	45.7% (16/35)	**0.005**
SS14	16.7% (5/30)	59.1% (13/22)	**0.003**
Nichols	9.1% (1/11)	23.1% (3/13)	0.60

Significant values are in bold.

This rise might reflect a change in the consumption of macrolide antibiotics in Argentina. However, data on antibiotic use in our country is limited: a study on the evolution of the purchase of antibiotic drugs from Argentine pharmacies between 2015 and 2017 found no substantial change in the purchase of macrolides^41^. Another explanation could be an increased veterinary use of macrolides, as is the case with other pathogens^42^, and/or increased travelling. More publicly available information on medical and veterinary uses of macrolides in Argentina would be necessary to shed light on the observed increase in macrolide resistance-associated mutations in circulating TPA strains.

Of the 99 swab samples collected from patients with clinical suspicion of syphilis, 28 were ruled out as syphilis-negative. Among the remaining 71 samples, DNA isolation was unsuccessful in 14 cases, while treponemal DNA amplification was unsuccessful in 11 cases. These failures in DNA isolation and amplification may be due to low amounts of material collected during swabbing, to the presence of PCR inhibitors or to DNA degradation between swab collection and storage at − 20 °C. Since the exact amount of time elapsed was not recorded for all samples, it is not possible to verify whether there is a correlation between time at room temperature and amplification failure.

In this study, no statistically significant associations were found between clade or allelic profile and patient characteristics. However, patients carrying Nichols-like strains tended to be slightly older than patients carrying SS14-like strains. A significant association between the Nichols clade and older patients has been reported in clinical samples from France^43^. The Nichols clade also tended to be associated with higher VDRL titers, but this association has not, to our knowledge, been found in other studies.

## Conclusions

Molecular typing studies of human pathogenic bacteria can help elucidate the evolutionary history and origin of old diseases such as syphilis^17^, while also contributing to the understanding of transmission mechanisms, epidemic diversity and dynamics, as well as to the improvement of clinical practice and public policies of control and prevention^44^. Particularly in the case of TPA, a bacterium which has until recently^45^ resisted any attempt at long-term in vitro culture, molecular typing techniques prove especially useful. Global TPA typing data has facilitated the characterization of syphilis outbreaks, the discovery of associations between subtypes and neurosyphilis, the surveillance of macrolide resistance, the distinction between re-infection and reactivation, and the comprehension of the geographical, temporal and population distribution of TPA.

However, in spite of all its benefits, only a limited number of studies from a few countries have focused on molecular typing of TPA since the emergence of the first typing assays, especially in South America^44^. This study attempts to fill this gap of knowledge. Our results suggests that the prevalence of circulating TPA strains in our region may differ considerably from those reported in other regions, while also changing over time. Although the SS14 clade was shown to be dominant, the prevalence of the Nichols clade was higher than in most countries, and higher than in our previous study. Frequencies of allelic profiles also differed considerably from those reported in other regions and in our previous report. Notably, the prevalence of macrolide resistance-associated mutations increased dramatically between studies; the causes of this rise are of importance to public health and warrant further investigation. In addition to this, future molecular epidemiology studies of syphilis should aim at providing useful information for the development of prevention and control programs, focusing for instance on the identification of at-risk populations and sources of infection, as well as the verification of links between certain subtypes and pathology.

## Methods

### Clinical samples

Clinical swab samples were collected between May 2015 and November 2019 from patients with suspected primary or secondary syphilis at an STI clinic in the city of Buenos Aires (*Programa de Enfermedades de Transmisión Sexual* –PETS-, *Hospital de Clínicas “José de San Martín”*, University of Buenos Aires, Argentina). Although it is possible to detect treponemal DNA in non-lesion samples, lesions tend to have a higher treponemal load and thus usually yield higher DNA amplification efficiencies, therefore swab samples were taken only from lesions^46^. Swab samples from genital, anal, oral or rectal lesions were taken by a trained dermatologist, without cleaning the lesion. Samples were handled as described previously^47–49^. The swab samples remained at room temperature for no more than a week before being stored at − 20 °C until DNA isolation.

Patients suspected of having syphilis based on clinical findings were diagnosed with laboratory tests that included the examination of lesion material by DFM and serological tests. Serology included VDRL, FTA-ABS and TPHA^50^. Patients were considered to have syphilis when one of the tests (DFM and/or serology) was positive. Patients gave their written, informed consent and received recommendations of treatment, care, referral to the laboratory for serological diagnosis, and a new visit for follow-up. Clinical data included patients’ age, gender, STI history, location of lesion, results of serology and DFM, syphilis stage and HIV status. Socio-demographic data included residence, nationality, occupation and marital status.

### DNA isolation

DNA isolation from swabs was performed using a commercial kit (QIAamp DNA Blood Mini Kit, Qiagen, Germany). First, swabs were submerged in 600 µl of phosphate buffered saline. The eluate was then processed according to the manufacturer’s recommendations. The efficacy of the extraction process was verified by PCR amplification of the human beta-actin gene. The PCR mixture (final volume of 25 μl) contained 16.3 μl of water, 0.5 μl of a 10 mM deoxynucleotide triphosphate (dNTP) mixture, 2.5 μl of 10 × buffer, 3 μl of 25 mM MgCl_2_, 0.75 μl of each primer (10 μM), 0.25 μl of *Taq* DNA polymerase (5000 U/ml), and 1 μl of isolated total DNA. PCR amplification was performed under the following conditions: 95 °C (2 min); 94 °C (30 s), 60 °C (30 s), 72 °C (30 s) for 35 cycles; and 72 °C (7 min). Primers used can be found in Supplementary Table S1 online.

### Detection of treponemal DNA

In order to screen for TPA-positive samples, amplification of loci TP0105 (*polA*) and TP0319 (*tmpC*) was performed by nested PCR, as described previously^20^. The PCR mixture (final volume of 25 μl) contained in the first step 15.3 μl of water, 0.1 μl of a 10 mM dNTP mixture, 2.5 μl of 10 × buffer, 1.5 μl of 25 mM MgCl_2_, 0.25 μl of each primer (10 μM), 0.1 μl of *Taq* DNA polymerase (5000 U/ml), and 5 μl of isolated DNA. PCR amplification was performed under the following conditions: 94 °C (1 min); 94 °C (30 s), 58 °C (30 s), 72 °C (1 min) for 30 cycles; and 72 °C (10 min). The mixture for the second step was the same except that it contained 19.3 μl of water and 1 μl of PCR product from the first step. PCR amplification was performed under the following conditions: 94 °C (1 min); 94 °C (30 s), 58 °C (30 s), 72 °C (1 min) for 40 cycles; and 72 °C (10 min). Samples were considered PCR-positive for treponemal DNA when PCR amplification was positive for at least one treponemal locus. Primers used can be found in Supplementary Table S1 online.

### PCR amplification for MLST

MLST was performed to type all TPA-positive samples as described previously^14,15,43,51^. Four loci, including TP0136, TP0548, TP0705 and 23S rDNA genes, were amplified using the nested PCR protocol (both copies of the 23S rDNA locus were amplified). The PCR mixture (final volume of 25 μl) contained in both steps 20.5 μl of water, 0.5 μl of a 10 mM dNTP mixture, 2.5 μl of 10 × buffer, 0.25 μl of each primer (100 μM), 0.05 μl of *Taq* DNA polymerase (5000 U/ml), and 1 μl of isolated DNA. In the first step, PCR amplification was performed under the following conditions: 95 °C (1 min); 94 °C (30 s), 50 °C (30 s), 72 °C (1 min, 45 s) for 40 cycles; and 72 °C (7 min). In the second step, PCR amplification was performed under the following conditions: 95 °C (1 min); 94 °C (30 s), 55 °C (30 s), 72 °C (1 min, 45 s) for 40 cycles; and 72 °C (7 min). The second amplification step was repeated an additional three times, in order to quadruple the volume of PCR product per sample prior to DNA purification. PCR products were purified with polyethylene glycol (PEG), using an in-house protocol (see Supplementary Information). Primers used can be found in Supplementary Table S1 online.

### DNA sequencing and sequence analysis

Sequencing reactions were performed on the purified PCR products using a commercial kit (BigDye™ Terminator v3.1 Cycle Sequencing Kit, Applied Biosystems, USA). The products were sequenced on an automated capillary DNA sequencing system. Sequence analyses were performed using Sequencher software (Sequencher® Version 5.4.6 DNA Sequence Analysis Software, Gene Codes Corporation, Ann Arbor, MI, USA). TP0136 and TP0548 sequences were aligned to reference sequences TPA Nichols (CP004010.2) and TPA SS14 (CP004011.1) in order to assign samples to the corresponding clade (clade assignment was possible where at least one of these loci was successfully sequenced). 23S rDNA sequences were evaluated at positions 2058 and 2059 in the 23S rDNA gene of *Escherichia coli* (accession no. V00331), where A → G mutations are associated with macrolide resistance. Alleles encoding resistance were marked A2058G or A2059G depending on the site of substitution. Both copies of the rDNA gene were sequenced and analysed.

### Phylogenetic analyses

Clade assignment was confirmed through phylogenetic analysis: the software MEGA X^52^ was used to construct multiple sequence alignments for each gene separately via the ClustalW algorithm^53^, as well as for a concatenation of TP0136, TP0548 and TP0705 sequences, which were then used for the construction of phylogenetic trees, using the maximum likelihood method^54^ with bootstrapping (1000 replications, cutoff value of 50%)^55^ and the Tamura-Nei model. MLST of TPA is based on the different allelic profiles (haplotypes) described by a 3-letter code, where the first number corresponds to the TP0136 allele, the second to the TP0548 allele, and the third to the TP0705 allele (e.g. 1.3.1).

### Statistical analyses

Comparisons were performed using Fisher's exact test, for categorical variables, and the Mann–Whitney U test, for numerical variables, via the SPSS software (IBM Corp. Released 2011. IBM SPSS Statistics for Windows, Version 20.0. Armonk, NY: IBM Corp).

### Ethics statement

This study was approved by the Ethics Committee of the *Hospital de Clínicas “José de San Martín”* (University of Buenos Aires). All patients gave their written, informed consent. The study was conducted according to the principles expressed in the Declaration of Helsinki^56^, as well as national regulations regarding research.

## Supplementary Information

Supplementary Information.

## Data Availability

All alleles found in this study were identical to previously reported sequences, which can be found in the PubMLST database^15^.
