# Supplementary material for: High frequency of Nichols-like strains and increased levels of macrolide resistance in Treponema pallidum in clinical samples from Buenos Aires, Argentina
Source: Sci Rep. 2022 Sep 29;12:16339. doi: 10.1038/s41598-022-20410-5 (PMC9522787; doi:10.1038/s41598-022-20410-5)
Supplement: Supplementary file 1 — Supplementary Information. [file 41598_2022_20410_MOESM1_ESM.pdf]

High frequency of Nichols-like strains and increased levels of macrolide resistance in *Treponema pallidum* in clinical samples from Buenos Aires, Argentina.

Nicolas Morando[1], Eliška Vrbová[2], Asunta Melgar[3], Roberto Daniel Rabinovich[1], David Šmajs[2], María A. Pando[1,*]

[1]CONICET-Universidad de Buenos Aires. Instituto de Investigaciones Biomédicas en Retrovirus y Sida (INBIRS), Buenos Aires, Argentina.

[2]Department of Biology, Faculty of Medicine, Masaryk University, Brno, Czech Republic.

[3]Universidad de Buenos Aires, Hospital de Clínicas "José de San Martín", Programa de Enfermedades de Transmisión Sexual (PETS), Buenos Aires, Argentina.

*mpando@fmed.uba.ar

Supplementary Methods. Protocol for PCR product purification with polyethylene glycol.

PCR products were purified using polyethylene glycol (PEG), according to an in- house protocol. Briefly, 1.1 µl of PEG solution (PEG 0.2 g/ml, NaCl 0.15 g/ml) were added to 1 µl of PCR product, mixed and incubated for 15 minutes at 37°C. Then, centrifuged at 15.000 g at room temperature for 15 minutes. After adding 3.8 µl of cool 80% ethanol per µl of PCR product, the mixture was incubated one minute at room temperature and the supernatant was discarded. The DNA was left to dry at 37°C for 30 minutes and finally suspended in 25 µl of molecular-grade water.

Supplementary Table S1. Primers used for nested-PCR

Locus	External primers (5'-3')	Coordinates[a]	Length of PCR product (bp)	Internal primers (5'-3')	Coordinates[a]	Length of PCR product (bp)
human beta-actin	AGCGCAAGTACTCCGTGTG[b]	-	107	-	-	-
	CGGACTCATCGTACTCCTGCTT[b]	-		-	-	
TP0105	TTCTGTGCTCACGTCTGGTC	-	637	TGCGCGTGTGCGAATGGTGTGGTC	-	376
	TGCAACCATCGTATCGAAAA	-		CACAGTGCTCAAAAACGCCTGCACG	-	
TP0319	CTGCTCATCGGCTGCTCTA	-	773	GAAGGTGGTGACTTCGTCGT	-	451
	ACCACAGACTTCGACCCATC	-		CAAAACCCGCTTCAAAGAGA	-	
TP0136	AACCCGTTAGCGCCCAACAT	157804-157823	1789	AGTGTCTTCCTCGTCCGTTC	158206-158225	1206
	TCCCAGCTCAGCCGAATCTC	159570-159589		CACGTGGTGGTGTCAAACTT	159392-159411	
TP0548	TGGGGCACTAAACCGGAAGA	593136-593155	1567	GCGGTCCCTATGATATCGTGT	593285-593305	1065
	TACGGGCATTTGCGGATAGG	594683-594702		GAGCCACTTCAGCCCTACTG	594330-594349	
TP0705	GGTCTATATGCAGCCCTTCTTC	772663-772684	1181	TGCGGCTTATCCTGATGAATAG	772917-772938	803
	GCTTGAGAACGATACCGGATAC	773822-773843		TATTCTGCGGCGTTGGATAG	773700-773719	
23S rDNA[c]	CGAAGGGAAGCAGGTGTAGT	234704-234723, 283149-283168	1666 and 1658	GTACCGCAAACCGACACAG	234768-234786	629
	GCGCGAACACCTCTTTTTAC	236350-236369		AGTCAAACCGCCCACCTAC	235378-235396	
	GAACCGTCCCTGAAAACTCA	284787-284806				

[a] Acoording to Nichols genome (CP004010.2). Coordinates are only included for typing loci.

[b] A single pair of primers was used for beta-actin amplification.

[c] Both copies of 23S rDNA gene were amplified.
